# Tensor-Based Morphometry and Stereology Reveal Brain Pathology in the Complexin1 Knockout Mouse

**DOI:** 10.1371/journal.pone.0032636

**Published:** 2012-02-29

**Authors:** Catherine Kielar, Stephen J. Sawiak, Paloma Navarro Negredo, Desmond H. Y. Tse, A. Jennifer Morton

**Affiliations:** 1 Department of Pharmacology, University of Cambridge, Cambridge, United Kingdom; 2 Wolfson Brain Imaging Centre, Addenbrooke's Hospital, Cambridge, United Kingdom; 3 Behavioural and Clinical Neuroscience Institute, University of Cambridge, Cambridge, United Kingdom; Institute of Psychology, Chinese Academy of Sciences, China

## Abstract

Complexins (Cplxs) are small, soluble, regulatory proteins that bind reversibly to the SNARE complex and modulate synaptic vesicle release. Cplx1 knockout mice (*Cplx1^−/−^*) have the earliest known onset of ataxia seen in a mouse model, although hitherto no histopathology has been described in these mice. Nevertheless, the profound neurological phenotype displayed by *Cplx1^−/−^* mutants suggests that significant functional abnormalities must be present in these animals. In this study, MRI was used to automatically detect regions where structural differences were not obvious when using a traditional histological approach. Tensor-based morphometry of *Cplx1^−/−^* mouse brains showed selective volume loss from the thalamus and cerebellum. Stereological analysis of *Cplx1^−/−^* and *Cplx1^+/+^* mice brain slices confirmed the volume loss in the thalamus as well as loss in some lobules of the cerebellum. Finally, stereology was used to show that there was loss of cerebellar granule cells in *Cplx1^−/−^* mice when compared to *Cplx1^+/+^* animals. Our study is the first to describe pathological changes in *Cplx1^−/−^* mouse brain. We suggest that the ataxia in *Cplx1^−/−^* mice is likely to be due to pathological changes in both cerebellum and thalamus. Reduced levels of Cplx proteins have been reported in brains of patients with neurodegenerative diseases. Therefore, understanding the effects of Cplx depletion in brains from *Cplx1^−/−^* mice may also shed light on the mechanisms underlying pathophysiology in disorders in which loss of Cplx1 occurs.

## Introduction

Complexins (Cplxs) are small, soluble, regulatory proteins [1] that bind reversibly to the SNARE complex, playing an important role in the modulation of neurotransmitter release [2], [3] [4] [5] and [6]. Recently, these proteins have been shown to have a dual function, both suppressing tonic vesicle release and promoting stimulus-evoked release [7]. This dichotomy is achieved by the specific binding of different domains of the protein to different regions of the SNARE complex [8] [9] and [10].

Four different Cplx isoforms have been identified so far [1]. The two major brain isoforms, Cplx1 and Cplx2 [2], are highly homologous in mammals [11] and [1]. In the brain, all neurons express one or other isoform of Cplx, furthermore Cplx1/Cplx2 double knockout mice die at birth, suggesting that Cplxs play an essential role in the brain. Morphological studies showed that Cplx1 and Cplx2 are present in neuronal cell bodies, processes, and synapses [1] and [12]. In the mouse brain Cplx1 and Cplx2 have a largely reciprocal expression [13] and [14] and have been reported to be selectively enriched in GABAergic terminals and glutamatergic terminals respectively in the cerebellum [15] and [12].

Despite the wide interest in the molecular actions of Cplxs, the importance of different isoforms in the physiology of brain function is unclear. Mice with a single isoform knocked out show very different phenotypes from each other. In particular, Cplx1 knockout (*Cplx1^−/−^*) mice have a pronounced ataxia [16], [17] and [18] whereas *Cplx2^−/−^* mice have subtle progressive deficits in motor, cognitive and social behaviours [16] and [19]. In *Cplx1^−/−^* mouse synapses there is a decrease of neurotransmitter release [2], although another study suggests that there is likely to be an optimal amount of Cplx in the synapse, since the overexpression of Cplx2 also inhibits neurotransmitter release from normal PC12 cells [20]. In addition to abnormal phenotypes of *Cplx1^−/−^* mice, there is indirect evidence showing that abnormal Cplx distribution or expression is present in a number of neurological diseases. Abnormal Cplx expression patterns have been found in patients and animal models of psychiatric and neurodegenerative disorders, such as Huntington's Disease (HD) [21], schizophrenia [22], [23] and [24], depression [22], [25] and [26], bipolar and unipolar disorder [27], [28], [29] and [30], Alzheimer's disease [31], Parkinson's disease [32], foetal alcohol syndrome [33] and [34], alcoholism [35] and [34] and traumatic brain injury [36].

The dominant phenotype in *Cplx1^−/−^* mice is a severe ataxia, which is present by 2 weeks of age [2]. These mice also have profound exploratory and emotional deficits [17] and [37]. In humans, ataxia is known to be associated with disturbances affecting the cerebellar output either via abnormalities in the cerebellar circuitry or in any of the constituent neurons or cells in those circuits [38], [39], [40] and [41]. The majority of mouse models of ataxia (reviewed in [42], [43] and [44] exhibit profound cerebellar degeneration. In contrast, the ataxia of *Cplx1* ^−/−^ mice has been reported in the absence of degeneration [2] and [11].

We have recently used whole brain automated morphometry based on MRI in a mouse model of HD (R6/2 line) to reveal a number of changes in the R6/2 mouse brain that were not apparent from using manual morphometry [45] and [46]. Therefore, we decided to use this method to investigate differences in the brains of *Cplx1* ^−/−^ mice. We found a number of significant differences in brain volume. We investigated those most likely to be related to the behavioural phenotype in greater detail, using stereological analysis with histology.

## Results

### MRI predicts an increased probability of atrophy of the thalamus and cerebellum and hypertrophy of white matter and neostriatum in Cplx1^−/−^ mice

**Figure 1:**
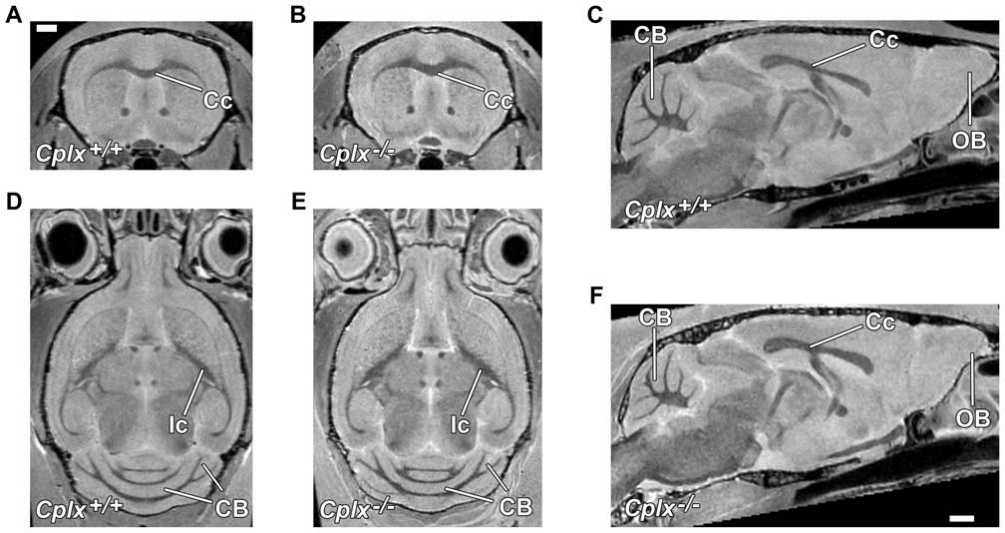
MRI reveals volume changes between *Cplx1^−/−^* and *Cplx1^+/+^* mice. Coronal (A, B), horizontal (D, E) and sagittal (E, F) MR images of *Cplx1^−/−^* and *Cplx1^+/+^* mouse brains. Volume changes can be seen in the corpus callosum (Cc), the olfactory bulbs are smaller (OB), internal capsule (Ic) and cerebellum (CB).

There was no difference in overall brain volume between *Cplx1^−/−^* and *Cplx1^+/+^* mice as measured by total intracranial volume (*Cplx1^−/−^* 488±16 mm^3^, *Cplx1^+/+^* 491±14 mm^3^). However, comparison of the MRI sections themselves revealed a number of clear differences between the brains (Fig. 1). Note that, in this figure, the two brains have been rigidly aligned without changing the size of any structure, to facilitate visual inspection. *Cplx1^−/−^* mice appear to have thickened white matter structures including the corpus callosum and the internal capsule and the olfactory bulbs appear to be smaller (Fig. 1). In the cerebellum, some of the lobes also appear to be smaller.

**Figure 2:**
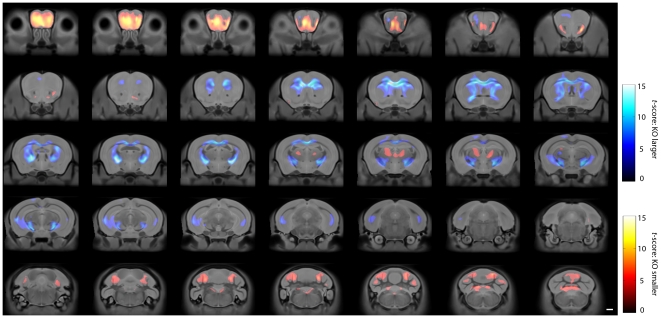
Tensor-based morphometry analysis of the volume changes between *Cplx1^−/−^* and *Cplx1^+/+^* mice. Coronal sections showing heat maps with significant volume differences between the *Cplx1^−/−^* and *Cplx1^+/+^* brains. The scale bar shows the significant level (*t*-score 26 degrees of freedom). To indicate the directionality of the tests for ease of interpretation, a separate colour mapping is used for areas of apparent atrophy (red colour scale) vs. hypertrophy (blue colour scale).

**Figure 3:**
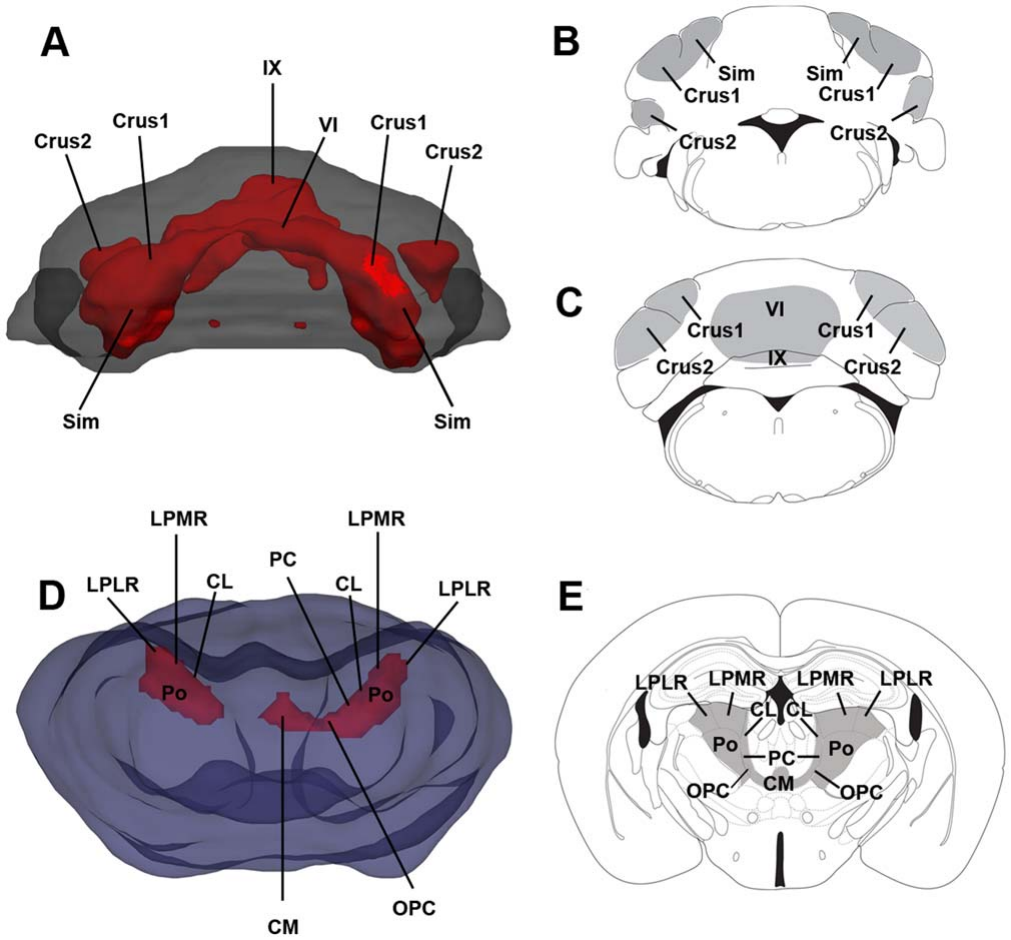
Atrophy in the cerebellum and thalamus in *Cplx1^−/−^* mice. 3D reconstruction maps of tensor-based morphometry showing the regions of atrophy (red) in the cerebellum (A) and thalamus (D), and 2D cartoons showing coronal sections of the cerebellum (B, C) and thalamus (E) showing the same regions of atrophy (grey). (Sim) simple lobule, (Crus1) Crus1 of the ansiform lobule, (Crus2) Crus2 of the ansiform lobule, (VI) lobule VI, (IX) lobule IX, (CL) centrolateral nucleus, (CM) central medial nucleus, (LPLR) lateral posterior nucleus laterorostral, (LPMR) lateral posterior nucleus mediorostral, (Po) posterior thalamic nuclear group, (PC) paracentral nucleus and (OPC) oval paracentral nucleus.

TBM of the MRI revealed areas of statistically significant volume differences between some regions in *Cplx1^−/−^* and *Cplx1^+/+^* brains. There was an increased volume of the central white matter including the corpus callosum, internal capsule, cerebral peduncles and external capsule, particularly the lateral aspects bordering the hippocampal formation. TBM of MRI also revealed a reduction in grey matter volume in the olfactory bulbs, the thalamus and cerebellum (Fig. 2). In the thalamus, the volume loss was particularly clear in the ventrolateral and centromedial nuclei, namely the centrolateral nucleus (CL), the central medial nucleus (CM), the lateral posterior nucleus (LP), posterior thalamic nuclear group (Po) and the paracentral (PC) and oval paracentral nuclei (OPC) (Fig. 3). The atrophy of the cerebellum also appears to be selective, particularly affecting lobes VI (simple lobule, Crus1 ansiform lobule), VII (Crus2 ansiform lobule) and IX. A full table of findings from the TBM analysis is given in a table as Table S1.

### Stereology shows no change in volume of caudate putamen and corpus callosum but confirms a decrease in thalamic and cerebellar volumes in Cplx1^−/−^ mice

**Figure 4:**
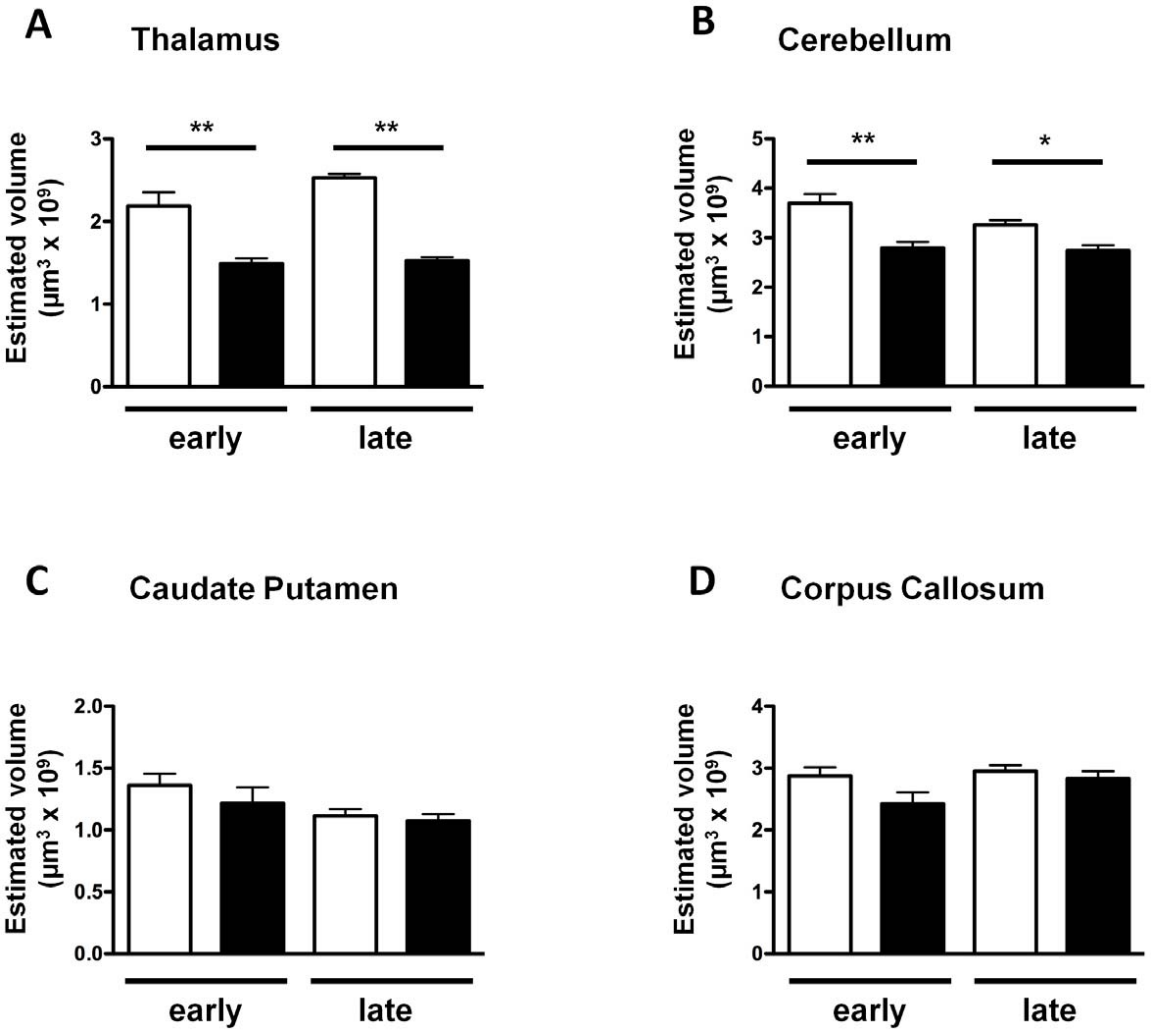
Region-specific atrophy in the *Cplx1^−/−^* mouse brain. Volumes of thalamus (A), cerebellum (B) caudate putamen (C) and corpus callosum (D) were measured at early and late stages in *Cplx1^+/+^* (open columns) and *Cplx1^−/−^* mice (closed columns). (* p<0.05; ** p<0.01; *** p<0.001, all columns show means ±SEM).

Volumes were estimated using stereology for *Cplx1^−/−^* and *Cplx1^+/+^* mice at two age points, one ‘early’ the other ‘late’. The histology-based estimates did not show any significant difference between the volume of the corpus callosum and the caudate putamen at either early or late stages (Fig. 4C, 4D). (Note that the lack of significance was not unexpected in the corpus callosum, given that central white matter structures in the brain are not well suited to volume measurements in techniques requiring serial sectioning.) In contrast, the volume of the cerebellum and thalamus was significantly smaller in the *Cplx1^−/−^* animals when compared to the *Cplx1^+/+^* at both ages (Fig. 4A, 4B). No significant changes in volume were seen between mice killed at the early and late stages. This suggests that, as with the behavioural phenotype, the pathological phenotype is non-progressive. Similarly, no significant differences in volume were observed between the *Cplx1^+/+^* early and late mice, suggesting that no age-dependent brain volume changes occur in *Cplx1^+/+^* animals over this period.

### Region specific atrophy in the cerebellum of Cplx1^−/−^ mice

**Figure 5:**
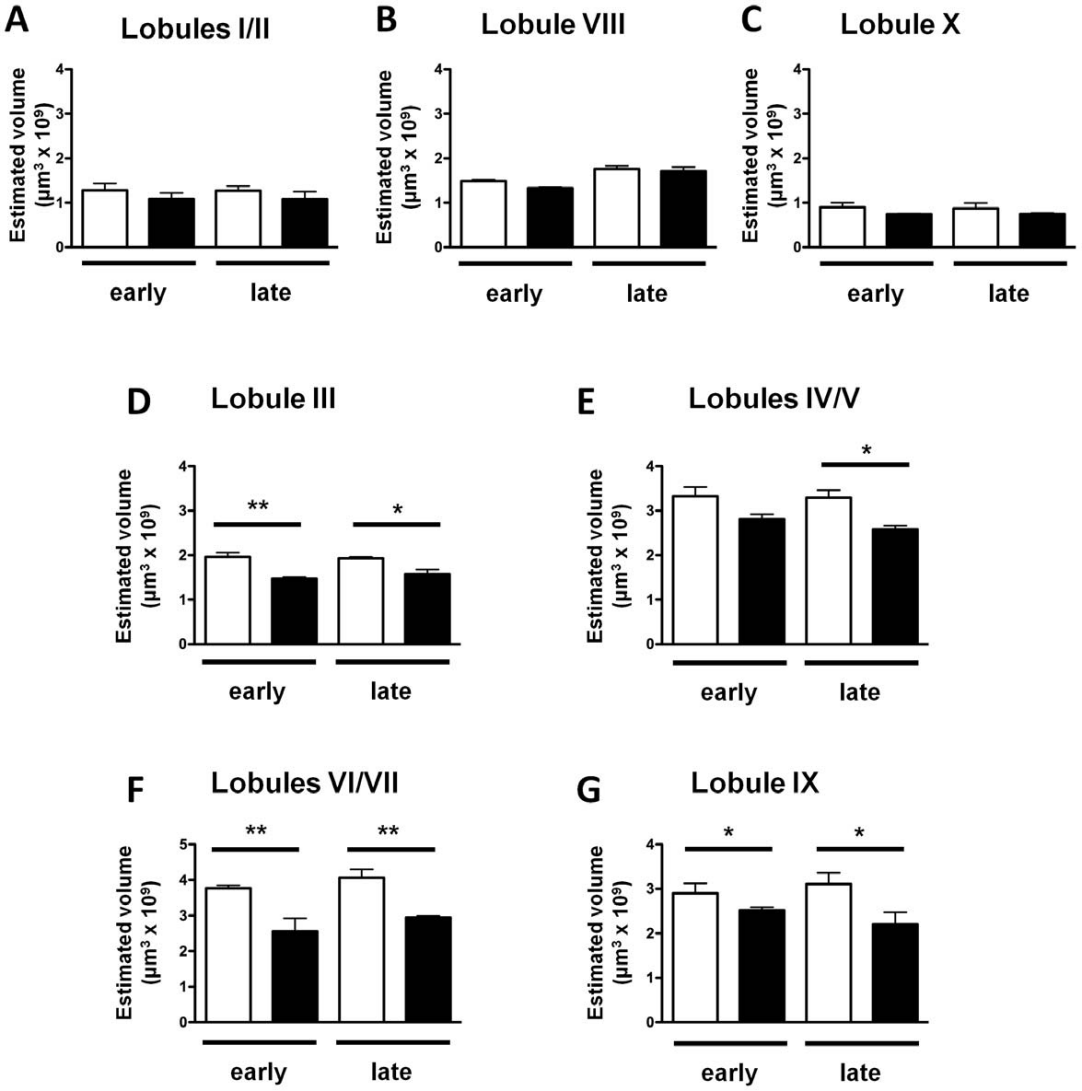
Atrophy occurs in specific cerebellar lobules in *Cplx1^−/−^* mice. Volumes of lobules I/II (A), VIII (B), X (C), IV/V (D), IX (E), III (F) and VI/VII (G) were measured at early and late stages in *Cplx1^+/+^* (open columns) and *Cplx1^−/−^* mice (closed columns). (* p<0.05; ** p<0.01; *** p<0.001, all columns show means ±SEM).

Since both our TBM and stereological data supported the idea that there was a decrease in the volume of cerebellum, and this was likely to be relevant to the ataxic phenotype of the mice, a more detailed study was conducted in this region. No significant differences between genotype or age were observed in the volume of cerebellar lobules I/II, VIII or X (Fig. 5A, 5B and 5C). By contrast, a clear difference was observed in lobules III (Fig. 5D), VI/VII (Fig. 5E) and IX (Fig. 5G) with volumes of *Cplx1^−/−^* mice being significantly smaller than those of *Cplx1^+/+^* mice, both at an early and a late stage. A similar trend was observed for lobule IV/V, but only the late time point reached statistical difference (p<0.05) (Fig. 5F). Thus, using stereology, we confirmed that *Cplx1^−/−^* mice have a reduced cerebellar volume in some of its individual lobules. To investigate the cause of these differences in *Cplx1^−/−^* mice, a more detailed histological study in lobules III, IV/V, VI/VII and IX was conducted.

### Atrophy of the molecular layer and neuron loss in the granule layer in the cerebellum of Cplx1^−/−^ mice

**Figure 6:**
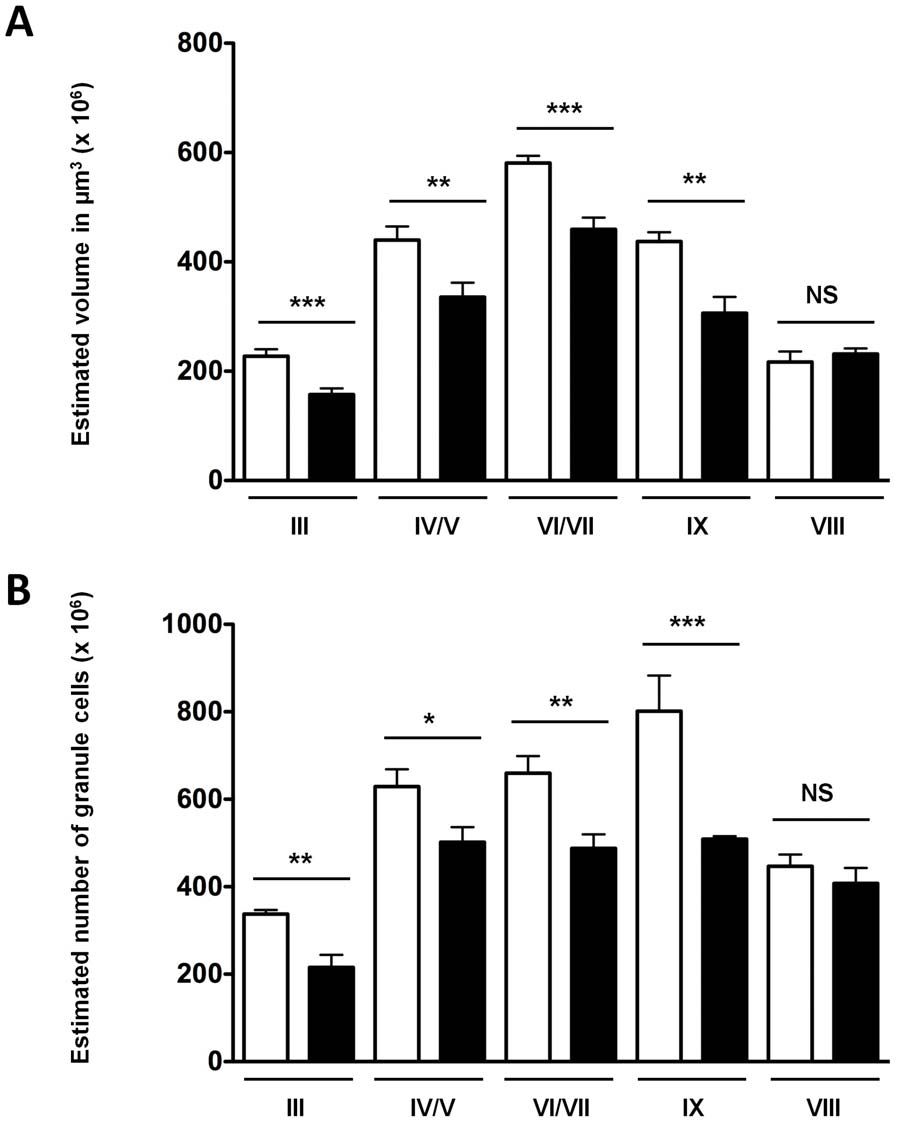
Decrease in volume of molecular layer and neuron loss in the granule layer in cerebellar lobules III, IV/V, VI/VII, VIII and IX in *Cplx1^−/−^* mice. Volumes of lobules III, IV/V, VI/VII IX and VIII (A) were measured at late stage in *Cplx1^+/+^* and *Cplx1^−/−^* mice. Numbers of Nissl stained granule cells in cerebellar lobules III, IV/V, VI/VII, IX and VIII were measured at late stage in *Cplx1^+/+^* and *Cplx1^−/−^* mice (B). (* p<0.05; ** p<0.01; *** p<0.001, NS not significant, all columns show means ±SEM).

Cplx1 expression in the cerebellar cortex was found in both cells and terminals of the Purkinje cell layer and stellate and basket cells of the molecular layer [47]. To determine whether the selective volume loss observed in the cerebellum of *Cplx1^−/−^* mice was due to the atrophy of the molecular layer, volume measurements were performed for those lobules that previously displayed a significantly reduced volume in *Cplx1^−/−^* animals, i.e. lobules III, IV/V, VI/VII and IX. We chose to analyse volume rather than thickness of the layer because of the great variability in the shape of the lobules, which might have an impact on accuracy, reliability and significance of this measure. Small but significant differences in the volume of molecular layer were found for all lobules studied (III, IV/V, VI/VII, IX); (Fig. 6A).

The reduction of molecular layer thickness might be indicative of neurodegeneration. Therefore, we decided to document the extent of neuronal loss in those lobules, using unbiased stereology. Although it seemed intuitively that atrophy of the molecular layer was due to cell loss, no significant loss of neurons was evident in the molecular layer of lobule III (Fig. 6B), IV/V, VI/VII and IX (data not shown) in late stage *Cplx1^−/−^* and *Cplx1^+/+^* mice. However, the molecular layer contains relatively few neurons and consists mainly of neuropil including extensions of apical dendritic tufts of pyramidal neurons and horizontally-oriented axons from granule cells, as well as glial cells [48]. To investigate the possibility that neuropil loss might be due to loss of axons/processes from granule cells, we used unbiased stereology to examine the number of cells in the granule cell layer in atrophic cerebellar lobules. Significant loss of granule neurons was found in lobules III, IV/V, VI/VII and IX (Fig. 6B) in late *Cplx1^−/−^* mice when compared to *Cplx1^+/+^* mice. We also quantified neuronal numbers in a lobule that did not reveal volume changes (lobule VIII). We found no significant difference in neuron numbers in this lobule between *Cplx1^−/−^* and *Cplx1^+/+^* mice (Fig. 6B).

## Discussion

When *Cplx1^−/−^* mice were first described by Reim *et al.* [2], no significant macroscopic changes in the structure of their brains, the distribution of synaptic markers or levels of other synaptic proteins were reported. We have also never seen abnormalities in macroscopic brains structure or distribution of neuronal markers (e.g. calcium binding proteins) that suggest otherwise (AJM unpublished data). Nevertheless, the profound neurological phenotype displayed by these mutants suggests that significant functional abnormalities must be present in these animals. Here we used, MRI with TBM, an approach that has proved useful in automatically detecting regions where structural abnormalities exist [49]. We show for the first time that the volume of thalamus and cerebellum was significantly smaller in *Cplx1^−/−^* animals when compared to controls. It would have been impracticable to look for small anatomical abnormalities by assessing every area of the brain using a traditional histological approach. However we were able to show that the reduced volume of the cerebellum and thalamus in *Cplx1^−/−^* mice found using MRI was confirmed through finer resolution analysis using unbiased stereology. Having also used TBM to good effect in picking up abnormalities in the R6/2 mouse model of Huntington's disease [45] and [46], we suggest that TBM of MRI is a powerful tool that allows the detection of small anatomical changes in brains that do not appear anatomically abnormal.

In this article, we focus our discussion on the changes affecting the grey matter. However, the white matter differences shown here by the TBM suggest that further studies using diffusion tensor imaging (DTI) would be appropriate, since DTI can be used for probing structural integrity of white matter fibres *in vivo* [50] and has proved a sensitive marker in a range of conditions [51].

The appearance of a decreased volume in specific brain regions of the *Cplx1^−/−^* animals at the early stage and its non-progressive nature suggests that the loss of Cplx1 affects the early development of these animals.*Cplx1^−/−^* mice present with a very early motor phenotype (ataxia from postnatal day 7; [19]). In the cerebellum, granule cell proliferation and migration [52] and [53] as well as growth of cerebellar afferents both occur during early post-natal development [54] and [55], with neurons in the cerebellum reaching their final position at approximately postnatal day 15 in mice [56]. There is some evidence that modulation of circuit formation [57], [58] and [59] and cytodifferenciation [60] in the brain is controlled by neurotransmitter release during development. Since a decrease of neurotransmitter release has been found in *Cplx1^−/−^* mice [2], it is therefore possible that early developmental abnormalities could occur due to abnormalities in neurotransmitter release that leads to a dysgenesis of the cerebellum. Further studies will be needed to determine the precise cause of the pathological abnormalities described here.

High levels of Cplx1 are expressed in both thalamus and cerebellum [14] and [47]. Although we did not focus on the thalamus in the detailed histological analysis, our MRI results show that show that the thalamic volume changes in *Cplx1^−/−^* mice affect specific nuclei. This is interesting, given that the thalamus is a routeing station for all incoming sensory impulses, and that each thalamic nucleus is specific for the type of information it receives [61]. Some of the thalamic nuclei showing atrophy are important for relaying modalities that are impaired in *Cplx1^−/−^* mice. For example, the centrolateral nucleus, lateral posterior nucleus and the posterior thalamic nuclei group, all relay motor information [62] and [63], and motor coordination is severely impaired in *Cplx1^−/−^* mice. Interestingly however, some thalamic nuclei showing atrophy are important for relaying modalities that have not been examined in *Cplx1^−/−^* mice. For example, the centrolateral nucleus relays noxious visceral information [64], and the central medial nucleus is involved in nociception [65], [66] and [67] as well as seizure activity [68] and [69]. The paracentral and oval paracentral nuclei, are involved in nociceptive processing related to the affective and motivational aspects of pain [65], [66] and [67]. It would be interesting to see if *Cplx1^−/−^* mice have deficits relating to abnormal processing of those modalities.

It is well known that the cerebellum plays a major role in motor coordination and the fine adjustment of movements (for review see [70]), and abnormalities in cerebellar development and/or function often result in a typical uncoordinated phenotype in mice (for review see [71]). In addition, previous studies have shown cerebellar atrophy in other mouse models of ataxia [72], [73] and [71]. Therefore, although pathological abnormalities in the cerebellum have not been reported in *Cplx1^−/−^* mice previously, it was not surprising to find significant volume loss in the cerebellum of these mice. However, it was interesting that the volume loss in the cerebellum was not generalized but rather that lobes were differentially affected, with some lobes apparently spared in *Cplx1^−/−^* mice. Although the division of the cerebellum into lobules is based on anatomical distinctions, and as yet no specific functions have been attributed unequivocally to each of the lobules, some of them are very well known for being involved in specific functions. For example, in cats lobules VI–VII denominated the “oculomotor vermis” are important in the control of eye movements [74]. A topographic organization of motor control and cognitive and emotional processing in the cerebellum has also been hypothesized [75]. As well, the role of the cerebellum in human cognition has been the focus of recent reviews [76], [77]. According to this hypothesis, the anterior lobe of the cerebellum (lobules I–V) plus parts of lobule VI and Lobule VIII constitute the sensorimotor cerebellum, while lobule VIII and parts of lobule VI form the cognitive cerebellum. Therefore, changes in the volumes of lobules III and IV/V, which are involved in motor control, may contribute to the motor phenotype seen in *Cplx1^−/−^* mice, while the sensory [78] and social [17] deficits seen in these animals may be due to changes in lobules VI/VII, that we have shown to be smaller in the *Cplx1^−/−^* mice.

Detailed analysis of volume loss in the cerebellum revealed a significant loss of granule cells, as well as a reduction in molecular layer volume. The reduced volume of the molecular layer was not due to reduced number of cells but was likely to be due to a loss of processes from granule cells that send processes in the molecular layer [79]. A loss or degeneration of these processes could directly induce granule cell degeneration. As said previously, it is not clear if the cell loss in *Cplx1^−/−^* mice is due to developmental abnormalities or neurodegenerative processes; either of these is possible. Since Cplxs are centrally important for controlling Ca^2+^-triggered exocytosis [80], a depletion of Cplxs will have deletrious effects on synaptic transmission and neurons lacking Cplxs show dramatically reduced transmitter release efficiency due to decreased Ca^2+^ sensitivity of the synaptic secretion process [2]. Reduced neurotransmitter release can cause mal-formation, or no formation at all, of synapses during development [58] and [59]. A similar phenomenon might cause a reduced neuron number in *Cplx1^−/−^* mice. It is also possible that a reduced cell number in *Cplx1^−/−^* mice could be due to excitotoxicity caused by a lack of GABAergic inhibition. Indeed, although it is not the case in all parts of the brain, in the cerebellum, Cplx1 is selectively expressed in inhibitory neurons [12]. Loss of Cplx1 would lead to a decrease in inhibition and could result in an increased activity of granule cells and could lead to cell death via excitotoxic mechanisms. A loss of granule cells due to the increased glutamate transmission observed in the Purkinje cell/parallel fibre synapse has been found in several mouse models of ataxia [81], [82] and [83]. In addition to granule cells, the granule layer is also composed of Golgi cells, which provide an inhibitory feedback to the granule cells [79]. A decrease in inhibitory pathways could again lead to excitotoxic damage. Loss of both granule and Golgi cells has been observed in the ‘leaner’ mutant mouse, which displays severe ataxia and cell loss in the cerebellar cortex [84]. In summary, dysgenesis and neurodegeneration are two possible outcomes of Cplx1 deficiency that results in ataxia in *Cplx1^−/−^* mice. For example, in the Weaver mouse model granule cells cannot migrate and differentiate into the granule layer [85], whereas the SCA1 and SCA2 mouse models display pure neurodegeneration [86] and [87]. However, no studies have been conducted in Cplx1−/− mice at embryonic stages and we cannot be certain as to which mechanism is involved in the pathology in those mice.

It is well documented that the majority of ataxias (for review see [88]) as well as patients with Wernicke-Korsakoff syndrome [89] show cerebellar atrophy. The involvement of the thalamus has also been reported previously in patients with spinocerebellar ataxias type 2, 3 and 7 [90], [91], [92] and [93], autosomal recessive ataxia [94] and patients with Wernicke-Korsakoff syndrome [89]. In addition, loss of granule cells in the cerebellum has been previously reported in siblings with ataxia-telangiectasia-like disorder [95] as well as in mouse models of ataxia [84], [82] and [83]. Although the relationship between Cplx1 and ataxia is not known [2] and [11], the high levels of Cplx1 expression in the cerebellum and thalamus [14], the reduced volume of these regions in *Cplx1^−/−^* mice reported here and the involvement of the cerebellum and thalamus in movement control seem to indicate that both these regions might be involved in the development of the abnormal motor function that is a result of Cplx1 depletion in mice.

Our study is the first to describe pathological changes in the cerebellum and the thalamus of the *Cplx1^−/−^* mouse brain. The cerebellar and thalamic regions that show atrophy in *Cplx1^−/−^* mice are important for motor as well as cognitive processing. Therefore, it seems likely that pathology in these regions contributes to ataxia and possibly other phenotypic abnormalities observed in the *Cplx1^−/−^* mouse. Our current understanding of the role of changes in Cplxs in neurodegenerative diseases is poor. A better understanding the effects of Cplx depletion in the brain may shed light on its role, not only in the normal brain but also in the mechanisms underlying pathophysiology in ataxias and neurodegenerative disorders in which loss of Cplxs occurs.

## Methods

### Animals

Cplx1 mice were generated by homologous recombination in embryonic stem cells [2]. All *Cplx1* ^−/−^ mice used in this study were F1 or F2 mice bred from heterozygote mice on a mixed genetic background (129Ola/C57Bl6) in a colony established in the Department of Pharmacology, University of Cambridge. Cplx1 mice have since been backcrossed onto a C57/Bl6 inbred background for 10 generations, without change in the overt phenotype (unpublished observations). All experimental procedures were licensed and undertaken in accordance with the regulations of the UK Animals (Scientific Procedures) Act 1986.

For MRI, thirty adult mice were used; n = 12 *Cplx1^+/+^* (age range 50–52 mean 51 weeks) and n = 18 *Cplx1^−/−^* (age range 34–78 mean 59 weeks). The brains of R6/2 mice were scanned *ex vivo* because their health status did not allow the study to be done *in vivo*. Animals were deeply anaesthetized with Avertin (10 ml/kg i.p.) and perfusion fixed. Brains were not removed from the skull in order to preserve overall brain shape and to avoid damage to structures adjacent to the pial surface. Specimens were stored in 4% paraformaldehyde solution under refrigeration until they were imaged.

For stereological volume measurements another cohort of mice was used. Brains from an ‘early’ (aged between 4 and 10 weeks) and a ‘late’ (>2 years of age), group of *Cplx1* ^−/−^ mice and age-matched littermate controls were harvested. In *Cplx1* ^−/−^ mice the ataxic gait is apparent by postnatal day 7 (P7) and is pronounced by the time of weaning (21 days) [17], therefore it is important to note that both ‘early’ and ‘late’ groups of animals are symptomatic. Mice were killed for histology by asphyxiation with rising levels of CO_2_ (n = 4 at each age and genotype). Brains were removed and immediately frozen on dry ice. For stereological neuron quantifications, an additional ‘late’ group (n = 3) of *Cplx1* ^−/−^ mice and age-matched littermate controls were perfused transcardially with 4% paraformaldehyde in 0.1 M phosphate-buffered saline (PBS) and immersion fixed for at least 1 week in 4% paraformaldehyde. All brains were subsequently cryoprotected in a solution of 30% sucrose in PBS until required.

#### Animal husbandry

All mice were housed in hard-bottomed polypropylene experimental cages in groups of 10–16 mice. Lighting was controlled on a 12 h light: 12 h dark cycle. The housing facility temperature was maintained at 21–23°C and the relative humidity was also controlled (55±10%). Clean cages were provided twice weekly with corncob bedding and fine shredded paper nesting material. The mice had *ad libitum* access to water and standard dry laboratory food. As *Cplx1^−/−^* mice have difficulty in balance rearing, all *Cplx1^−/−^* mice were provided with lowered waterspouts and twice daily supplementary feeding. Supplementary feeding consisted of softened chow pellets placed on the floor of the home cage twice daily.

#### Genotyping

Genotyping was done using DNA prepared from tail biopsies and carried out using a polymerase chain reaction (PCR) as described previously [17].

### MRI

To assess the morphological phenotype of *Cplx1^−/−^* mouse brains, we imaged the brains using high-resolution MR microscopy and used tensor-based morphometry (TBM), a method that localizes differences in the local shape of brain structures, to evaluate the differences between genotypes [49].

Immediately prior to imaging, specimens were immersed in Fluorinert FC-70 (3 M, Inc.), a proton-free susceptibility-matching fluid to reduce wraparound artefacts due to fixation fluid whilst preventing artefacts due to the magnetic field inhomogeneities due to air boundaries close to the brain. Imaging was performed using a Bruker PharmaScan 4.7 T system with a 20 mm birdcage resonator for sample excitation and signal reception.

A rapid acquisition with relaxation enhancement (RARE) sequence was used (repetition time (TR)/echo time (TE) 2000/32 ms, echo train length (ETL) 8, number of excitations (NEX) 2), with matrix 256×192×128 over field of view 17.9×13.4×9.0 mm^3^ yielding an isotropic resolution of 70 µm in a scan time of 3.5 h). These parameters were chosen to optimise contrast between grey and white matter in the brain.

Images were processed using SPM5 (Wellcome Trust Centre for Neuroimaging, UCL) [96] with the SPM Mouse toolbox [45] and [46] (http://www.wbic.cam.ac.uk/~sjs80/spmmouse.html). Brains were manually adjusted for approximate alignment with the tissue probability maps from our previous studies of the mouse brain [45] and [46]. Unified segmentation [96] was used to perform affine registration, bias correction and non-linear registration to bring the brains into the same stereotactic space and produce maps of grey matter and white matter.

These maps were used with DARTEL [97], a diffeomorphic registration algorithm to accurately register the brains. The Jacobian determinants at each voxel within the brain were calculated using the deformation fields from the DARTEL algorithm and smoothed using an isotropic Gaussian kernel of 400 µm. To compare whole brain volumes, the total amounts of GM, WM and fluid in the brain from the segmented tissue maps were summed to give total intracranial volume for each specimen.

### Stereology

Serial sections (30 or 50 µm) were cut serially through the whole brain of each mouse, making sagittal sections of the cerebellum and coronal sections of the rest of the brain. The sections were then mounted onto gelatinised slides in order to be processed for histochemical staining. Slides were subsequently stained with Cresyl violet (Sigma Chemical Company, Dorset, UK). Slides were defatted in Histoclear (Cellpath, Powys, Mid Wales, UK) for 3 min, followed by dehydration for 2 min each in absolute ethanol and 95% ethanol, and 1 min in 70% ethanol. The sections were then washed with clean tap water for 30–60 s. After soaking in 1% Cresyl violet for 15 min, slides were again washed with tap water for 30–60 s until the water ran clear. They were then differentiated for 10 s in 1% acetic acid/alcohol and washed in distilled water. After 1 min in 70% ethanol, slides were placed for 2 min in each of 95% then absolute ethanol twice. Samples were placed in three successive pots of Histoclear for 1, 3 and 3 min, respectively, and coverslipped with DPX mounting medium (BDH, Lutterworth, Leicestershire, UK). After mounting the slides were dried in an oven at 37°C for at least 24 hours.

Unbiased Cavalieri estimates of the volume [98] of the caudate putamen, corpus callosum, thalamus, cerebellum, cerebellar lobules and cerebellar layers were made on Nissl stained sections using a Nikon Eclipse 80i microscope and StereoInvestigator software (Micro-brightfield Inc., Williston, VT, USA), with the operator blind to genotype. An appropriately spaced sampling grid (250 µm thalamus; 200 µm caudate putamen and cerebellum; 125 µm corpus callosum and cerebellar lobules IV/V and VI/VII; 100 µm cerebellar lobules I/II, III, VII and IX; 75 µm cerebellar lobule X; 50 µm cerebellar molecular layer) was superimposed over the sections and the number of points covering the relevant areas counted using an ×2 objective. Regional volumes expressed in µm^3^ were collected for each animal and the mean volume of each region obtained for *Cplx1^+/+^* and *Cplx1^−/−^* mice. The delineation of each region was consistently performed by referring to the Paxinos and Franklin Mouse Brain Atlas (2004) [99].

To examine neuronal survival within the molecular layer of individual cerebellar lobules we used *StereoInvestigator* software to obtain unbiased optical fractionator estimates of neuronal numbers in Nissl stained sections. These estimates were obtained for lobules III and VIII. These measures were performed with a random starting section chosen, followed by every fifth Nissl stained section thereafter. For lobule III, the counting frame size was 20 µm×20 µm and the grid size used was 150 µm×120 µm. For lobule IV/V, the counting frame size was 20 µm×20 µm and the grid size used was 200 µm×120 µm. For lobule IX, the counting frame size was 20 µm×20 µm and the grid size used was 150 µm×120 µm. Finally, for lobule VI/VII, the counting frame size was 20 µm×20 µm and the grid size used was 200 µm×150 µm.

### Statistical Analysis

For MRI, measures of total intracranial volume (mean ± SD) were compared with a two-tailed Student's *t*-test with *P*<0.05 used as a threshold for statistical significance. A general linear model was fitted to the smoothed Jacobian determinant values at each voxel for genotype with sex and overall brain volume as covariates. The effect of genotype was assessed at each voxel as a two-sample Student's *t*-test. To control for multiple comparisons, the false-discovery rate (FDR) was controlled at *q*<0.01 [100]. With this correction one would expect, on average, that 1% of the results reported to be significant are in fact false positives. In addition to this, a cluster-extent threshold was applied, ignoring all clusters with a total number of voxels fewer than 500.

For stereology, a two-way ANOVA was used to analyze all the volume and quantifications data (mean ± SEM) with a *post-hoc* Bonferroni test. *P*<0.05 was considered as statistically significant. For all optical fractionator estimates, the mean coefficient of error of individual estimates was calculated according to the method of Gundersen and Jensen [99] and was <0.05 in all analyses.

## Supporting Information

Table S1 **Significant regions found in the TBM analysis between** ***Cplx1^+/+^*** **and** ***Cplx1^−/−^*** **mice.** Structures are reported at the centre of each cluster, which may extend into surrounding areas within and around the region denoted as the major structure (p<0.01 FDR-corrected, cluster extent greater 500 only). Coordinates are given relative to bregma (left–right, anterior-posterior and inferior-superior respectively).
(DOC)
