# Supplementary material for: Tensor-Based Morphometry and Stereology Reveal Brain Pathology in the Complexin1 Knockout Mouse
Source: PLoS One. 2012 Feb 29;7(2):e32636. doi: 10.1371/journal.pone.0032636 (PMC3290572; doi:10.1371/journal.pone.0032636)
Supplement: Table S1 — Significant regions found in the TBM analysis between Cplx1+/+ and Cplx1−/− mice. Structures are reported at the centre of each cluster, which may extend into surrounding areas within and around the region denoted as the major structure (p<0.01 FDR-corrected, cluster extent greater 500 only). Coordinates are given relative to bregma (left–right, anterior-posterior and inferior-superior respectively). (DOC) [file pone.0032636.s001.doc]

| **Cluster extent** | **Voxel pFDR** | **Voxel T** | **Bregma coordinates / mm** | | | **Structure** | **Major structure** |
| --- | --- | --- | --- | --- | --- | --- | --- |
| Areas where Cplx1^-/-^ < Cplx1^+/+^ | | | | | | | |
| 41426 | 1.7x10 -8 | 12.94 | 0.35 | 3.43 | -2.10 | External plexiform layer | Olfactory bulb |
|  | 1.7x10 -8 | 12.64 | -0.98 | 4.06 | -1.54 | Granular cell layer | Olfactory bulb |
|  | 1.7x10 -8 | 12.13 | 0.42 | 4.55 | -1.54 | External plexiform layer | Olfactory bulb |
| 14118 | 2.3x10 -7 | 9.02 | -1.54 | -6.93 | -1.68 | Crus1 ansiform lobule | Cerebellum |
|  | 1.2x10 -6 | 8.04 | -1.75 | -6.44 | -2.31 | Cerebellum | Cerebellum |
|  | 2.3x10 -6 | 7.66 | 1.96 | -6.09 | -2.52 | Simple lobule | Cerebellum |
| 654 | 5.0x10 -7 | 8.54 | 2.87 | 1.33 | -4.48 | Lateral olfactory tract | Central white matter |
| 5278 | 4.2x10 -6 | 7.35 | 0.77 | -7.21 | -3.71 | 9th Cerebellum lobule | Cerebellum |
|  | 5.4x10 -6 | 7.22 | -0.49 | -7.28 | -3.64 | 9th Cerebellum lobule | Cerebellum |
|  | 1.5x10 -5 | 6.72 | 1.54 | -7.42 | -3.85 | 9th Cerebellum lobule | Cerebellum |
| 4431 | 1.5x10 -5 | 6.74 | -0.98 | -2.03 | -3.08 | Posterior thalamic nuc | Thalamus |
|  | 1.9x10 -4 | 5.55 | 0.98 | -1.89 | -2.87 | Posterior thalamic nuc | Thalamus |
|  | 6.4x10 -4 | 4.99 | 1.61 | -2.45 | -1.96 | Posterior thalamic nuc | Thalamus |
| 872 | 1.8x10 -4 | 5.58 | -2.73 | -6.86 | -3.01 | Crus2 ansiform lobule | Cerebellum |
|  | 2.9x10 -3 | 4.28 | -2.94 | -6.23 | -3.43 | Crus2 ansiform lobule | |
| Areas where Cplx1^-/-^ > Cplx1^+/+^ | | | | | | | |
| 96343 | 3.0x10 -9 | 14.63 | 0.77 | 0.70 | -1.68 | Corpus callosum | Central white matter |
|  | 3.0x10 -9 | 14.22 | 1.89 | -0.84 | -3.85 | Internal capsule | Central white matter |
|  | 3.0x10 -9 | 13.56 | -2.10 | -1.05 | -3.85 | Internal capsule | Central white matter |
| 1798 | 2.3x10 -5 | 6.44 | 0.91 | 3.01 | -1.75 | Intrabulbar ant commissure | Olfactory bulb |
|  | 2.7x10 -4 | 5.24 | 1.33 | 2.66 | -1.19 | Frontal associative cortex | Cortex |
|  | 4.8x10 -4 | 4.96 | 0.28 | 2.31 | -1.05 | Prelimbic cortex | Cortex |
| 2261 | 9.1x10 -5 | 5.74 | -3.99 | -3.92 | -3.22 | External capsule | Central white matter |
|  | 2.9x10 -4 | 5.19 | -3.78 | -3.78 | -3.78 | External capsule/Subiculum | Central white matter |
|  | 7.1x10 -4 | 4.78 | -3.71 | -3.99 | -2.45 | External capsule | Central white matter |
